# Three-Dimensional Evaluation of Alveolar Bone Levels Around Dental Implants and Natural Teeth: A Prospective Study

**DOI:** 10.7759/cureus.71129

**Published:** 2024-10-09

**Authors:** Kratee Sharma, Shweta Aggarwal, Kamakshi Gupta, Ravi Madan, Sahrish Tariq, Inderpreet S Narula, Seema Gupta

**Affiliations:** 1 Department of Periodontics, Kothiwal Dental College and Research Centre, Moradabad, IND; 2 Department of Periodontics, Institute of Dental Sciences, Jammu, IND; 3 Department of Prosthodontics, Kothiwal Dental College and Research Centre, Moradabad, IND; 4 Department of Public Health Dentistry, Swami Devi Dyal Hospital & Dental College, Panchkula, IND; 5 Department of Orthodontics, Kothiwal Dental College and Research Centre, Moradabad, IND

**Keywords:** alveolar bone, bone loss, cone-beam computed tomography, dental implant, periodontium

## Abstract

Introduction: Dental implants' efficacy is influenced by the quality and quantity of the osseous tissue present at the site designated for dental implants. Therefore, this study was conducted with the primary objective of assessing marginal bone loss around mandibular posterior dental implants at six and 18 months after prosthesis placement, compared with the contralateral natural tooth. The secondary objective was to determine the correlation between various factors such as periodontal status, age, gender, duration between first and second-stage surgery, and marginal bone loss.

Materials and methods: This prospective, split-mouth study was conducted on 24 patients, consisting of two groups: group 1, with single tooth replacement by a dental implant in the posterior mandible, and group 2, a contralateral natural tooth. The bleeding index (BI), probing depth (PD), and marginal bone loss were evaluated using cone-beam computed tomography at six and 18 months follow-up period after prosthesis placement.

Results: Significant bone loss was observed in both groups across all regions (p < 0.05). The mean marginal bone loss was greater at 18 months in group 1 than at six months of follow-up, whereas in group 2, it was not significant. The total mean marginal bone loss during the follow-up period of 18 months was 0.05 mm on the buccal and lingual side, whereas it was 0.5 mm on the mesial side and 2 mm on the distal side. Similarly, in the control group, the total mean marginal bone loss was 0.03 mm on the buccal and lingual sides, whereas it was 0.04 mm on the mesial and distal sides. The PD and duration of second-stage surgery were significant predictors and showed a significant correlation with marginal bone loss.

Conclusion: PD and marginal bone loss were significantly higher in the implant group than in the natural tooth group over the follow-up period of 18 months, particularly on the distal side.

## Introduction

In contemporary times, prosthetic restoration of dental structures has undergone significant transformation due to the advent of dental implants. Dental implants are constructed using titanium, which is recognized for its biocompatibility and durability. Numerous implant systems are currently available commercially. Dental implants represent synthetic tooth roots that are surgically placed within patients to serve as a reliable and enduring foundation for dental bridges or prosthetic teeth [1]. The success of dental implants depends on various factors, including the specific implant utilized, patient characteristics, and proficiency of the clinician performing the procedure. Among the patient-related factors, periodontal tissue status plays a critical role. The bone surrounding an implant is susceptible to resorption, and this peri-implant bone loss can significantly influence the ultimate results of the implantation process [2].

The extent of osseous resorption during the first year of implant functionality ranged from approximately 0.9 to 1.6 mm. Furthermore, a subsequent annual average osseous resorption of 0.05 to 0.13 mm is considered standard. The assessment of initial bone resorption is crucial as it provides clinicians with an essential framework for determining the need for corrective and preventive interventions [3]. According to a study by Aliabadi et al. [4], it was noticed that marginal bone loss around implants was minimal at three months of prosthesis placement and increased at six months of follow-up. Similarly, Velasco-Ortega et al. [5] conducted a study to evaluate marginal bone loss around implants at a mean follow-up period of 45 months and concluded that the mean marginal bone loss was 1.51 ± 1.16 mm during the follow-up. However, both studies evaluated bone loss using intraoral periapical radiographs (IOPAs), which have the inherent drawback of being two-dimensional. Currently, three-dimensional (3D) cone beam computed tomography (CBCT) is considered a very reliable tool for measuring bone levels [6]. Iqbal et al. [7] determined that the average bone resorption following the implantation period of one, two, and six weeks was 0.075 ± 1.432 mm, 1.542 ± 0.953 mm, and 1.049 ± 0.42 mm, respectively. However, the evaluation time was short.

Bacterial infections play a pivotal role in the failure of dental implants. Microbial communities associated with periodontitis and peri-implantitis exhibit considerable similarities. The integrity of healthy peri-implant tissue serves as a crucial biological barrier against various etiological agents responsible for peri-implant diseases; its compromise allows for direct bacterial invasion of the bone, precipitating rapid bone loss. Significant contributors to the onset and progression of peri-implantitis include excessive mechanical loading, suboptimal implant design, and corrosion that may arise when a non-noble metal component is affixed to a titanium implant [8]. Therefore, due to the lack of previous studies, this study was conducted with the primary objective of assessing the marginal bone loss around the mandibular posterior dental implants at six and 18 months after prosthesis placement, compared with the contralateral natural tooth. The secondary objective was to determine the correlation between various factors, such as periodontal status, age, sex, duration of second implant surgery, and marginal bone loss.

## Materials and methods

Study design

This prospective, case-control, slit-mouth study was conducted at the Department of Periodontology of the Kothiwal Dental College and Research Centre, Moradabad, India, from September 2022 to February 2024. Institutional ethics committee approval was obtained before starting the study (KDCRC/IERB/06/2022/16), and the study was conducted in accordance with the principles of the Declaration of Helsinki and Strengthening the Reporting of Observational Studies in Epidemiology (STROBE) guidelines. Written informed consent was obtained from all patients.

Sample size calculation

The requisite sample size was calculated using G*Power software version 3.6.9 (Universität Düsseldorf, Düsseldorf, Germany), employing an effect size of 0.36, which was derived from a preceding study [7], demonstrating a mean difference in buccal bone loss levels at different follow-up intervals around the single implant was 0.96 mm and a combined standard deviation (SD) of 2.67. A two-tailed statistical examination was performed to achieve a statistical power of 80% (β = 0.20) in conjunction with a significance level (α error) of 5%. Through the application of these criteria, a total sample size of 48 samples (24 participants for the split-mouth study) was considered sufficient to detect statistically significant differences between the groups.

Participants’ eligibility

Twenty-four patients who visited the department during the study period were selected for the study based on the following inclusion criteria: systemically healthy patients more than 18 years of age of both sexes, in whom the mandibular posterior implants were placed on one side for single tooth replacement with the contralateral natural tooth, presence of Angle’s class I occlusion with no parafunctional habits, opposing natural teeth on both sides, bone quality of D2 and D3 as determined by pre-treatment CBCT during the time of planning for implant placement [9], implant placement with a two-stage surgical procedure with a minimum two months of time period between the first and second stages, bleeding index (BI) < 1 [10], and probing depth (PD) < 3 mm [11]. Tobacco chewers, smokers, those with a history of bone disorders and alcohol consumption, use of corticosteroids, use of antibiotics in the past six months, radiation therapy, those with multiple missing teeth, edentulous patients, pregnant and lactating females, those with temporomandibular joint disorders, orofacial abnormalities, and those who needed guided bone regeneration around dental implants were excluded from the study.

Methodology and group allocation

In this split-mouth study, 48 teeth were evaluated in 24 patients by dividing them into two groups: group 1 (n = 24), in which single tooth replacement was performed in the posterior mandibular region by a dental implant, and group 2 (n = 24), in which the contralateral natural tooth acted as a control.

Group 1 had at least one single tooth replacement of the posterior tooth with a dental implant using a mechanical approach and a two-stage surgical procedure. The dental implants used were TSIII SA (Osstem Implant Co., Seoul, South Korea) of diameter 4.5 mm or 5.0 mm and length 8.5 mm or 10 mm (depending on bone availability). The surface of the implant underwent a combination of sandblasting and acid etching to enhance its roughness. This specifically defined treatment method resulted in a surface exhibiting increased roughness, with an average roughness (Ra) in the range of 1.5-2.0 µm. The implant was fabricated from commercially pure grade IV titanium, distinguished by its internal hexagonal connection. All implants were placed by a single operator with an experience of 10 years and specialized in implant dentistry (RM). After prosthesis placement, the BI and PD were recorded for all patients at the implant site and the control side using a sterile calibrated probe (UNC 15, Chicago, IL, USA). The indices were repeated at six and 18 months follow-up. All patients were instructed to undertake oral hygiene regimens using a manual toothbrush equipped with soft bristles and a non-abrasive toothpaste containing fluoride. Interdental brushes were recommended for meticulous cleaning of the areas surrounding implants. Furthermore, patients were strongly cautioned against the consumption of hard objects and ingestion of sticky food items.

**Figure 1:**
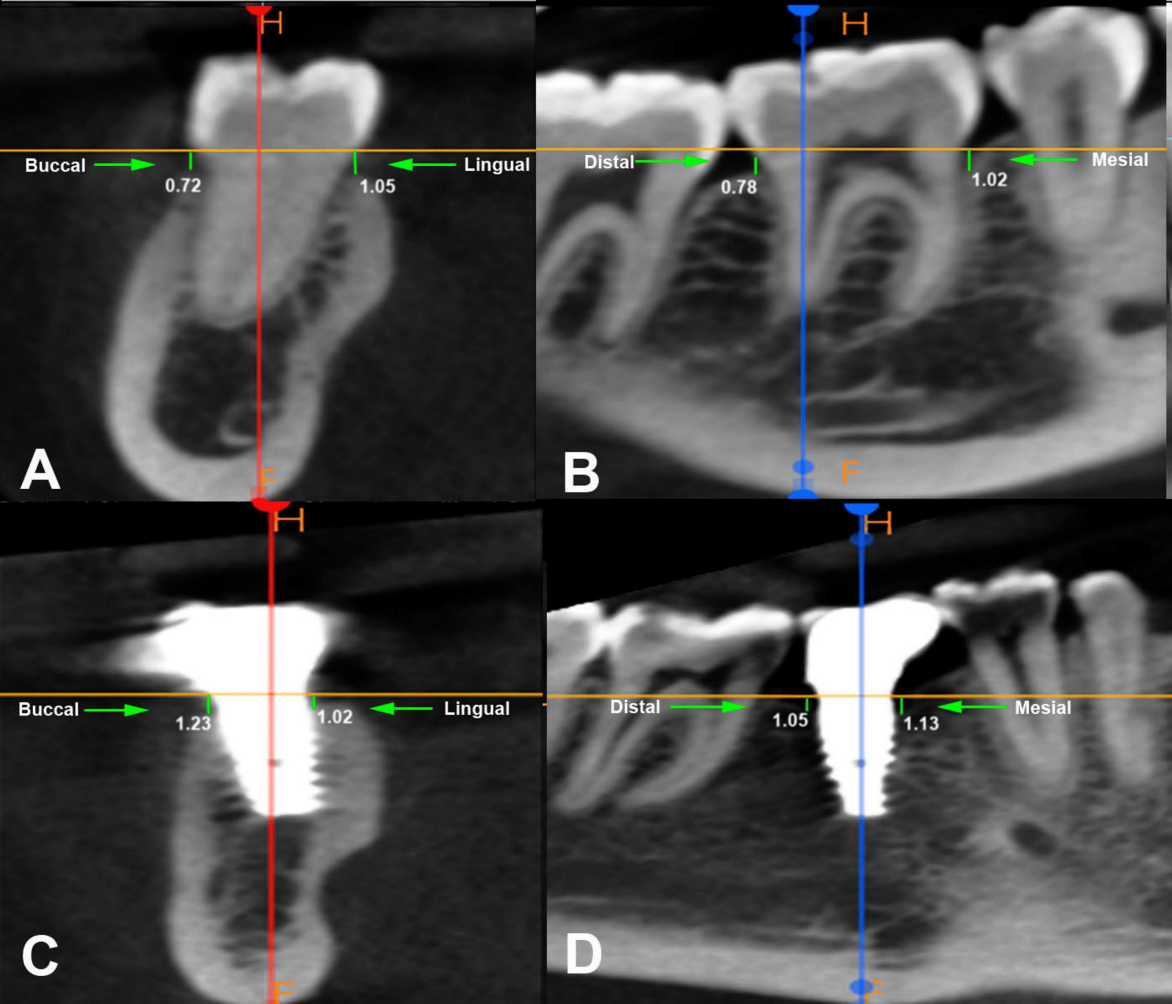
Evaluation of the marginal bone loss: (A) Buccal and lingual aspect of natural tooth, (B) mesial and distal aspect of natural tooth, (C) buccal and lingual aspect of dental implant, (D) mesial and distal aspect of dental implant.

CBCT scans were obtained for all patients at the six- and 18-month follow-up visits to estimate the marginal bone levels in groups 1 and 2. The bone measurements were assessed via CBCT with an accuracy of 0.1 mm using a CBCT apparatus (Carestream Dental CS 9600 CBCT, Kodak, Atlanta, Georgia) by a trained radiologist with over a decade of experience. The imaging was performed at a voltage of 85 kVp, with an average exposure duration of eight seconds at 4 mA, a grayscale depth of 15 bits, a voxel dimension of 0.2 mm, and a field of view spanning 5 × 5 cm. All imaging procedures were performed in alignment with the As Low As Reasonably Achievable (ALARA) guidelines to ensure minimal radiation exposure while maintaining the requisite diagnostic image quality. The subjects were positioned such that their Frankfort horizontal plane (a plane passing through the uppermost point of external auditory meatus to the lowermost point on bony orbit) was parallel to the floor, and efforts were made to minimize any movements that could result in image artifacts. Marginal bone loss was evaluated on the buccal, lingual, mesial, and distal sides as the distance from the cementoenamel junction (CEJ) to the crest of the alveolar bone (Figure 1).

Reliability

A single observer performed all periodontal measurements (KS), and three observers performed CBCT measurements (ST, KG, and SA). The intra-observer reliability for periodontal measurements was checked in randomly selected 10 individuals at two-day intervals using the intraclass correlation coefficient (ICC) and was found to be 92% ((ICC = 0.92; p < 0.001), showing high reliability and reproducibility. Similarly, intra- and inter-observer reliability was assessed on randomly selected 10 CBCT scans at three-week intervals by the same observers who were blinded to the previous measurements. The ICC values for the CBCT measurements showed excellent intra-observer agreement (ICC = 0.96; p < 0.001) and inter-observer agreement (ICC = 0.94; p < 0.001).

Statistical analysis

Data analysis was conducted using IBM SPSS Statistics for Windows, version 21.0 (released 2012, IBM Corp., Armonk, NY). Continuous variables are reported as mean and SD. The normality of the data was assessed using the Shapiro-Wilk test, which confirmed that the data followed a normal distribution. As the data were continuous and normally distributed, parametric tests were employed for inferential statistics. A one-way independent t-test and paired t-test were used to evaluate the results. Specifically, the independent t-test was used to assess the significance of alveolar bone loss in the groups (dental implant and contralateral natural tooth), while the paired t-test was used to examine bone loss during the 18-month interval within the group. Multivariate linear regression analysis was conducted to analyze the effect of independent variables on alveolar bone loss.

## Results

The descriptive analysis of the study sample revealed that the sample consisted of 14 (58%) males and 10 (42%) females, with a mean age of 35.42 ± 4.51 years. Sixteen (67%) patients were vegetarian. The duration of second-stage surgery was more than three months in 18 (75%) patients. The implant side was mostly on the left side in 16 (67%) patients, and all were mandibular first molars. The most commonly used implants were 10 mm and diameter 4.5 mm (Table 1).

**Table 1:** Descriptive analysis of independent variables. Data are presented in form of n (%).

Variables	Category	Frequency (n)	Percentage (%)
Gender	Male	14	58%
	Female	10	42%
Diet	Veg	16	67%
	Non-veg	8	33%
Duration between first and second-stage surgery (months)	≤3 months	6	25%
	≥3 months	18	75%
Implant side	Right	8	33%
	Left	16	67%
Implant length (mm)	8.5 mm	4	17%
	10 mm	20	83%
Implant diameter (mm)	4.5 mm	13	54%
	5 mm	11	46%

The comparison of marginal bone loss between dental implants and natural teeth during the six- and 18-month follow-ups was analyzed using a paired t-test. Significant bone loss was observed in both groups across all regions, with a p-value of 0.001 for buccal, lingual, mesial, and distal marginal bone losses. For group 1, Cohen's d values indicated a large effect size. Similarly, group 2 also showed significant bone loss with effect sizes of 0.99, 0.82, 1.14, and 1.02 for the buccal, lingual, mesial, and distal regions, respectively. This showed that at the 18-month post-prosthesis follow-up, the marginal bone loss was significantly greater in both groups, compared to the six-month follow-up (Table 2).

**Table 2:** Comparison of bone loss at six- and 18-month follow-ups by paired T-test. *p-value ≤ 0.05 is significant. Data are presented in the form of mean ± standard deviation (SD).

Groups	Parameters	Bone loss at six-month follow-up (mean ± SD)	Bone loss at 18-month follow-up (mean ± SD)	p-value	Effect size
Group 1	Buccal bone loss (mm)	1.33±0.10	1.38±0.10	0.001*	1.72
	Lingual bone loss (mm)	1.22±0.07	1.26±0.09	0.001*	1.58
	Mesial bone loss (mm)	2.19±0.20	2.26±0.18	0.001*	1.35
	Distal bone loss (mm)	2.25±0.19	2.30±0.19	0.001*	1.1
	Bleeding index	0.58±0.64	0.72±0.65	0.488	0.28
	Probing depth (mm)	2.05±0.58	2.34±0.24	0.028*	1.36
Group 2	Buccal bone loss (mm)	1.26±0.11	1.27±0.10	0.001*	0.99
	Lingual bone loss (mm)	0.93±0.87	0.94±0.09	0.001*	0.82
	Mesial bone loss (mm)	1.33±0.11	1.34±0.10	0.001*	1.14
	Distal bone loss (mm)	1.29±0.10	1.30±0.10	0.001*	1.02
	Bleeding index	0.45±0.49	0.51±0.45	0.660	0.18
	Probing depth (mm)	1.54±0.31	1.63±0.35	0.350	0.25

The comparison of various clinical parameters between the groups was performed using an independent t-test. No significant difference was found in the BI between the groups (p = 0.464). However, significant differences were observed in PD (mean difference = 0.51, p = 0.001), with group 1 showing increased PD. Buccal, lingual, mesial, and distal marginal bone losses also demonstrated significant differences between the groups. The mean differences were 0.04 mm for buccal bone loss (p = 0.001), 0.02 mm for lingual bone loss (p = 0.005), 0.06 mm for mesial bone loss (p = 0.001), and 0.03 mm for distal bone loss (p = 0.001), with all bone loss values being higher in group 1 compared to group 2. Maximum bone loss was observed on the distal surface compared to the other surfaces (Table 3).

**Table 3:** Comparison of different variables between both groups using independent t-test for a total follow-up period of 18 months. *p-value ≤ 0.05 is significant. SE: standard error. Data are presented in the form of mean ± standard deviation (SD).

Parameters	Group 1 (mean ± SD)	Group 2 (mean ± SD)	Mean difference	SE of difference	Lower limit	Upper limit	p-value
Bleeding index	0.05 ± 0.03	0.01 ± 0.01	0.13	0.17	-0.22	0.47	0.464
Probing depth (mm)	0.04 ± 0.02	0.02 ± 0.02	0.51	0.14	0.23	0.79	0.001*
Buccal bone loss (mm)	0.07 ± 0.05	0.01 ± 0.01	0.04	0.01	0.03	0.06	0.001*
Lingual bone loss (mm)	0.05 ± 0.04	0.01 ± 0.01	0.02	0.01	0.01	0.03	0.005*
Mesial bone loss (mm)	0.58 ± 0.65	0.46 ± 0.51	0.06	0.01	0.03	0.08	0.001*
Distal bone loss (mm)	2.05 ± 0.6	1.55 ± 0.32	0.03	0.01	0.02	0.05	0.001*

Multivariate linear regression analysis was used to examine the influence of various factors on the buccal, lingual, mesial, and distal marginal bone loss. Age and sex were not significantly correlated with bone loss in any region (p > 0.05). The duration between the first- and second-stage surgeries had a statistically significant effect on the mesial bone loss (p = 0.018); however, it was not significant in other regions. PD was a significant predictor of buccal bone loss (coefficient = 0.03168; p = 0.003). Diet and BI showed no significant relationship with bone loss in any of the regions. Overall, the results highlighted specific correlations between bone loss and factors such as duration between surgeries and PD, particularly for the lingual and mesial regions (Table 4).

**Table 4:** Multivariate linear regression analysis. *p-value ≤ 0.05 is significant.

Variables	Buccal bone loss		Lingual bone loss		Mesial bone loss		Distal bone loss	
Coefficient with p value	Coefficient	p value	Coefficient	p value	Coefficient	P value	Coefficient	p value
Age	0.00006	0.930	0.00054	0.409	-00205	0.878	0.00153	0.079
Gender	-16.55	0.264	1.61	0.897	1.134	0.90	7.78	0.550
Duration between first- and second-stage surgery (months)	-0.00018	0.979	-0.00423	0.502	0.03988	0.018*	0.00799	0.332
Diet	-17.31	0.249	9.75	0.491	10.18	0.363	6.51	0.649
Probing depth (mm)	0.03168	0.003*	0.00913	0.337	0.00076	0.943	0.01369	0.269
Bleeding index	-0.00314	0.713	-0.00767	0.326	-0.000003	0.997	-0.00859	0.397

**Figure 2:**
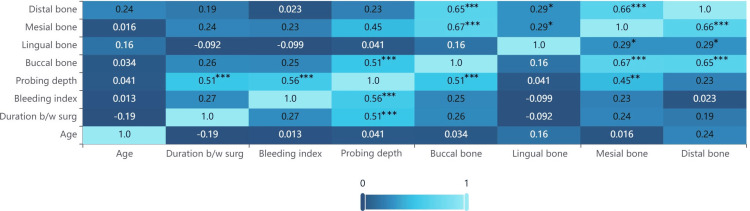
Pearson correlation heat map. *mild significance, **moderate significance, ***high significance. Very weak correlation: 0.0 < ∣r∣ < 0.20; weak: 0.2 ≤ ∣r∣ < 0.4; moderate: 0.4 ≤ ∣r∣ < 0.6; strong: 0.6 ≤ ∣r∣ < 0.8; very strong: 0.8 ≤ ∣r∣ ≤ 1.

The heat map displayed correlations between various clinical parameters, such as bone loss (distal, mesial, lingual, and buccal), PD, BI, age, and duration between surgeries. Strong positive correlations were observed between buccal, distal, and mesial bone losses, all of which were statistically significant (p < 0.05). PD showed a moderate correlation with both buccal bone loss and BI. In addition, buccal and lingual bone loss correlated moderately with mesial and distal bone loss. A weak correlation was observed between age and other variables, with age and mesial bone loss showing a low correlation of 0.016 (Figure 2).

## Discussion

The results of the present study indicate that the mean marginal loss was significantly greater in the implant group than in the natural tooth group over a follow-up period of 18 months. This finding is supported by previous studies [4,12,13]. The reason behind this observation could be because natural dentition is anchored to the osseous structure through the periodontal ligament (PDL), which functions as a cushioning mechanism, facilitating the even distribution of masticatory forces to the bone. Conversely, implants do not possess this ligament, resulting in the direct transmission of forces to the bone, potentially causing localized bone resorption if not appropriately addressed [14]. In contrast to natural dentition, which possesses the intrinsic ability to adapt to occlusal pressures, implants lack resilience, resulting in bone stress and subsequent resorption if the forces are inadequately apportioned [15].

Inadequate positioning, design, or dimensions of implants can result in increased stress on adjacent osseous structures. Nonideal implant angulations or insufficient osseous support can further exacerbate mechanical overload, leading to expedited bone resorption. Moreover, variations in bone density and quality may exist between the implant location and regions adjacent to the natural dentition [1]. Although these factors were not assessed in the current investigation, meticulous planning was undertaken using pre-treatment CBCT imaging, and all interventions were conducted by a single trained and proficient prosthodontist.

It was further revealed in the present study that PD was significantly correlated with marginal bone loss and was found to be greater on the implant side. This finding was supported by previous studies in which peri-implantitis was noted [8,12,16]. As all implants were stable over 18 months, significant peri-implantitis was not observed in our study, which could have led to non-significant changes in BI. The roughened surface of the implant to enhance osseointegration could have been a factor in this finding. The interface of an implant, especially at the convergence of the implant and crown, may facilitate bacterial colonization. If the abutment interface is not entirely sealed, it can result in a micro-gap that permits the accumulation of bacteria, ultimately contributing to inflammation in the surrounding soft tissues and subsequent bone resorption [16]. Dental implants are devoid of the PDL, which encases the natural dentition. The PDL provides a degree of resistance during probing, resulting in probing depths around the natural teeth that are typically shallower and more consistent. By contrast, the absence of a PDL barrier around implants allows deeper penetration of the probe into the surrounding soft tissues [14]. During implant insertion, a certain degree of initial osseous resorption or remodeling is anticipated as a component of the healing process. This phenomenon may result in augmented probing depths compared to natural dentition, which exhibits more stable osseous levels after eruption [1].

Our study indicated that marginal bone loss was greater on the distal surface, which could have been due to the fact that distal surfaces are more difficult to clean, leading to increased plaque accumulation. In addition, the anatomical structure of the alveolar bone located on the distal aspect of dentition or dental implants may exhibit a reduced thickness in comparison to other anatomical surfaces, thereby rendering it more susceptible to resorptive processes. In the posterior regions of the oral cavity, the bone quality, characterized by its density and volumetric attributes, frequently experiences a decline, which may increase the likelihood of resorption occurring within the distal bone [17].

The results of our study further indicated that the mean marginal bone loss was greater at 18 months in the implant group than at six months of follow-up, whereas it was not significant in the control group. This finding is in agreement with those of previous studies [12,13]. The total mean marginal bone loss during a follow-up period of 18 months was 0.05 mm on the buccal and lingual side, whereas it was 0.5 mm on the mesial side and 2 mm on the distal side. Similarly, in the control group, the total mean marginal bone loss was 0.03 mm on the buccal and lingual sides, whereas it was 0.04 mm on the mesial and distal sides. After a year of typical loading, Payne et al. observed a 0.35 mm loss in bone height at the crest; after two years, the bone loss was just 0.09 mm [18].

Our study further revealed that the duration of the time interval between the first- and second-stage implant surgery also had a significant correlation with marginal bone loss around the implants. More marginal bone loss was observed on the buccal and lingual sides if the loading time was less than three months, whereas more bone loss on the mesial and distal sides was observed if the loading time was more than three months. The ideal loading time for implants in the mandible is three to four months. If the duration of loading is less than three months, it is plausible that the osseous tissue may not have completely matured or integrated around the implant. In such instances, the buccal and lingual aspects, which are frequently thinner and more susceptible, may undergo a greater degree of marginal bone loss as a consequence of premature mechanical stress induced by functional forces (such as mastication) before full stabilization of the bone. The diminished cortical bone present on these surfaces is inherently more susceptible to resorption when subjected to mechanical stress before complete healing is achieved [19].

Clinical implications of our study

Given that bone loss and increased PD were significantly more pronounced within the implant cohort, especially in the distal aspect, it is imperative to conduct regular and frequent implant evaluations. This requires more frequent radiographic examinations (e.g., IOPAs or CBCT) and clinical probing to identify early indicators of bone loss and peri-implant pathology. Compared to natural dentition, implants may require more intensive monitoring, particularly during the initial 18-24 months following their loading. It may be advantageous to employ tissue grafts or guided bone regeneration methodologies to enhance osseous structures in distal regions during or after implant placement, particularly in individuals exhibiting inherently thin or compromised bone in these areas.

Limitations

The main limitation of our study was the lack of evaluation of bone levels at different levels of the alveolar crest. Moreover, only single implants in the mandible were evaluated in this study. Sex differences were not evaluated in our study, and many confounding factors could not be controlled, such as variation in angulation of the implant, differences in bone quality on the implant and control side, differences in maintenance of oral hygiene, and variations in implant height and thickness in the patients. Therefore, randomized controlled trials are required in this direction.

## Conclusions

Based on the findings of the present study, it was concluded that the mean marginal bone loss and PD were higher on the implant side than on the natural contralateral tooth during the 18-month follow-up period, and it was the highest on the distal side. Bone loss and PD were higher at the 18-month follow-up than at the six-month follow-up. The duration between the first- and second-stage implant surgery and PD were two significant predictors of bone loss at the mesial and distal sides of the implant. Age and sex were not significantly correlated with bone loss in any of the regions.
